# “Acting as the Face of a Broken System”: Challenges Experienced by Mobile Crisis Workers

**DOI:** 10.1007/s10597-026-01609-6

**Published:** 2026-04-06

**Authors:** Priya Gupta, Leah Jacobs, Kathryn Luk, Toya Jones

**Affiliations:** 1 Crown Family School of Social Work, Policy, and Practice, University of Chicago, Chicago, USA; 2 School of Social Work, University of Pittsburgh, Pittsburgh, USA

**Keywords:** Mobile crisis, Mental health, Workforce, Behavioral health, Crisis intervention, Community health

## Abstract

In 2020, United States federal legislation established the 988 Suicide & Crisis Hotline to divert mental health crisis calls away from standard emergency services, including law enforcement. The hotline’s creation has increased interest in and demand for comprehensive crisis intervention services. Among these services, mobile crisis (MC) teams are a core component. Expanding MC services, however, requires understanding the needs of and supporting the MC workforce. To inform these efforts, this qualitative study draws on first-hand perspectives on the challenges of MC work expressed by a national sample of MC workers (*n* = 364). Findings indicate MC workers experienced challenges across five primary domains; challenges related to structural characteristics of MC work, resources, the workplace and workforce, provider mental health, and behavioral health (BH) and criminal justice (CJ) systemic factors. Further, findings indicate that challenges related to the structural characteristics of MC work effected or were often compounded by issues pertaining to resources, workforce, mental health, and systems. Results revealed the need for improvements in several areas, including betterment of working conditions through implementation of a trauma-informed work environment, clarification of agency policies and procedures, development of training standards, increasing role clarity between MC workers and collaborators, and increased investment in community mental health resources and service systems.

In recent years, encounters between people experiencing mental and substance use-related crises (hereafter referred to as “behavioral health crises”) and police have received increased attention and high-profile media coverage, particularly those of disturbing, harmful and, at times, lethal nature (BHCs; see e.g., Borum et al., 1998; Levenson, 2021; *Pigeon v. City of Oklahoma City*, 2022; Saleh et al., 2018; Watson et al., 2008; Wood et al., 2017). In response to these harmful encounters and the increased burden of BHCs on traditional first response systems, United States federal legislation mandated a 3-digit call line for suicide and mental health crises (988; PL 116–172) in 2020. Since the law went into effect, many community mental health systems have made significant efforts to bolster crisis systems to respond to these calls (Fix et al., 2023; H.R. 1319, 2021). Despite significant cuts to federal funding for behavioral health services in the proposed 2026 budget, funds for crisis response explicitly remain stable (D.H.H.S., 2025), indicating rare bipartisan support for these services.

Mobile crisis (MC) teams, a community-based intervention that serves as a first-line response to BHC (Kisely et al., 2010), are a key component of comprehensive crisis response systems (Substance Abuse and Mental Health Services Administration [SAMHSA], 2020). MC teams vary in form but are typically comprised of some combination of clinicians, trained paraprofessionals, and peers (i.e., people with lived experience of mental illness trained to support others with mental illness) who travel to people experiencing BHCs in residences and other community settings. MC teams may operate directly with or alongside law enforcement, such as in co-response units comprised of MC workers and police. Typically, MC teams assess risk and safety, employ de-escalation techniques, and safety plan with service users (Fix et al., 2023). They may also make referrals to services, including supportive or involuntary services (e.g., involuntary hospitalization).

MC teams are unique, though they share characteristics with other mental health services and adjacent crisis response services (e.g., first responders; Bergen-Cico et al., 2020). Unlike other services, they are typically covert (i.e., operating as discretely as possible), involve working with a service user often with little background information, are deployed in the community, and occur almost exclusively during acute behavioral health problems. This means MC teams operate in contexts and under time constraints that increase pressure, complicate decision-making, and impact the ability to complete thorough psychosocial assessments and build rapport (Dyches et al., 2002; Helfgott et al., 2016).

Despite increased interest in crisis response and the uniqueness of MC teams, little is known about the MC workforce, the availability of adequately trained workers, or the factors that may sustain this workforce (Marcus & Stergiopoulos, 2022). In other words, existing literature is not clear on “what service providers need to best support individuals and communities” (Muehsam, 2019, p. 399), and a workforce perspective on MC services is largely absent from the empirical evidence base (Pietras & Wishon, 2021). Anecdotally, workforce prospects look grim; community mental health policymakers and program administrators bemoan a lack of qualified applicants and high turnover rates (see: Carroll et al., 2021; Waters, 2021), threatening the initiation of new MC programs, expansion of existing programs, and maintenance of program quality.

Research on related workforces and systems indicates a variety of potential problems for the MC workforce. Crisis response services are not uniform in their composition or structure and lack consistent funding for staffing and training (Bratina et al., 2021; Goldman et al., 2023; Pietras & Wishon, 2021), with staffing shortages exacerbated by burnout and vicarious trauma (Bailey et al., 2018; Bratina et al., 2021; Pietras & Wishon, 2021). These issues are a particular risk for MC teams because of the intense and frequent contact with people in severe crisis (Cunningham, 2003; Jones et al., 2022; Pearlman & Saakvitne, 1995a, 1995b), and are likely compounded due to worker frustration with limited resource and treatment options, particularly in rural coverage areas (Bailey et al., 2018; Bratina et al., 2021; Pietras & Wishon, 2021).

This study was designed to increase understanding of the challenges faced by MC team members by turning to MC providers themselves. Specifically, we draw on survey data from a national sample of MC team members (*n* = 364) to answer the following question: What are the greatest challenges MC team members experience in their work? In answering this question, we analyze how these challenges shape the experiences of MC workers and service delivery, and ultimately draw implications for workforce expansion, retention, and quality assurance.

## Methods

To understand the challenges MC workers face, we conducted a thematic analysis of open-ended questions from a national anonymous confidential survey of MC workers, following guidance from Terry et al. (2017), and secondarily from Miles and Huberman (1994). Though not without limitations (see Discussion), literature supports qualitative analysis of open-ended survey question responses as a particularly appropriate approach when the data reveal novel issues unknown during the conceptualization of the original study and when the content conveys strong emotional and substantive content, as is the case here (Boyatzis, 1998; Fereday & Muir-Cochrane, 2006; O’Cathain & Thomas, 2004).

## Sample

Because no single professional organization of MC workers exists and MC teams are located in a variety of private and public organizations, it is impossible to establish a universe of MC workers from which participants can be recruited. To promote recruitment of a sample that would reflect the diversity of MC workers nationally, we used a variety of strategies. Beginning in spring of 2023, we expanded on a previously developed list of organizations that housed MC teams and distributed recruitment materials to at least one organization in each state.^1^ We also distributed recruitment materials through a list serve hosted by Crisis Intervention Team (CIT) International, to organizations identified by practicum offices in schools of social work, and to professional contacts in community mental health services. Participants received $20 for completing the survey.

Recruitment yielded a sample of 364 participants who met inclusion criteria of being 18 years of age or older and currently working in mobile crisis response. The resulting sample included MC workers from 24 states across each of the five regions of the United States (Northeast, Southeast, Midwest, Southwest, and West). Participants were predominantly women (73.98%) and white (71.54%), had a mean age of 39.4 years (*SD* = 10.9), and 54.47% had a Master’s degree or higher. Most had a title of MC team member (67.48%) or MC supervisor (16.80%). While 36.59% of participants identified as a peer, only 10.30% had a job title of MC peer advocate. See Table 1 for a more detailed description of the sample. The sample demographically aligns with expectations, but CIT workers (45.2%) and supervisors (16.8%) may be overrepresented within the target population.

**Table 1:** Descriptive Statistics of Sample

	% (*n*)
Position Title	
MC team member	67.48 (249)
MC supervisor	16.80 (62)
MC peer advocate	10.30 (38)
Another position	4.61 (17)
**Age**	39.4 (10.9)*
**Gender**	
Non-Binary	2.71 (10)
Woman	73.98 (273)
Man	21.14 (78)
Another Gender	0.27 (1)
**Race**	
Black or African-American	8.13 (30)
Latine/x or Hispanic	10.57 (39)
Asian or Pacific Islander	2.17 (8)
White	71.54 (264)
Multi-racial	3.79 (14)
Another race	0.81 (3)
**Peer Identity**	
No	52.57 (194)
Yes	36.59 (135)
**SMI Diagnosis**	
No	74.80 (276)
Yes	18.70 (69)
**Education** **Level**	
Less than a Bachelor’s Degree	15.99 (59)
Bachelor’s Degree	22.22 (82)
Some Graduate School	6.78 (25)
Master’s Degree	46.88 (173)
More than a Master’s Degree	7.59 (28)
**Educational Background**	
Social Work	32.25 (119)
Counseling	22.49 (83)
Psychology	26.56 (98)
Marriage and Family Therapy	2.17 (8)
Other	22.22 (82)
**Tenure in Mobile Crisis Work**	
Less than 1 year	23.58 (87)
1–2 years	32.52 (120)
3–5 years	23.58 (87)
6–10 years	9.76 (36)
More than 10 years	10.30 (38)
**Tenure in Mental Health Work**	
Less than 1 year	5.69 (21)
1–2 years	16.80 (62)
3–5 years	23.85 (88)
6–10 years	21.14 (78)
More than 10 years	32.25 (119)

Note. Percentages may not add up to 100% due to rounding and exclusion of non-responses; * represents mean (standard deviation)

## Data Source

We drew data from an online survey conducted as part of a larger study on intervention decisions deployed during MC calls (e.g., de-escalation, engaging supports, and involuntary hospitalization petitions). In addition to sample characteristics, data for this study came from responses to two open-ended questions; *What is the most challenging thing about being a mobile crisis worker?* And *what helps or would help mobile crisis workers overcome this challenge?* These questions were intentionally developed to improve empirical understanding of challenges specifically faced by MC workers and to gain their perspectives on implementation of possible solutions. We treated responses to the first question as those of focus and used responses to the second question to verify our interpretation of responses to the first question, when related. Median responses were 145 characters in length (i.e., about the length of the last two sentences in this paragraph) and ranged from 6 to 1460 characters. The survey was completed with REDCap, a web-based survey administration platform (Harris et al., 2009, 2019).

## Analytic Procedures and Authenticity Assurances

To understand MC worker perspectives on the challenges of MC work, we analyzed participant responses with a 5-step thematic analysis (Terry et al., 2017). First, the first and second authors independently reviewed all text responses and discussed their impressions, developing a preliminary understanding of their content. Next, the first author inductively applied descriptive labels to the text and generated a preliminary list of codes. Both the first and second authors coded each response, iteratively refining codes together, applying the final codes to all responses, discussing any discrepancies, and resolving those discrepancies throughout. Thirdly, we generated themes of challenges expressed, organizing and grouping them into domains based on relationships and visually assessing patterns following guidance from Miles and Huberman (1994). Fourth, we finalized domain definitions and names, as well as dimensions within each domain. Finally, we reviewed our domains, eliminating domains not centrally related to the study’s aim (e.g., “personal problems”), clarifying the distinction between domains and the relationships between them, and tabulating counts for domains and dimensions.

To enhance transparency and authenticity, the first and second authors engaged in reflexive practices throughout data analysis, the team used researcher triangulation, and the third and last authors leveraged their clinical practice-based knowledge as a form of data authenticity (Charmaz, 2006; Ramalho et al., 2016). With respect to positionality, the authors are all women who are currently students or academicians, each identifies with unique ethnoracial groups (South Asian and white, white, East Asian, Black), and all had prior experience working in community mental health, including behavioral health crisis, with the last two authors having substantial experience in this area. The second author has also conducted non-participant observation of mobile crisis teams. These perspectives provided helpful insights into the underlying meaning behind survey responses. We supplemented this focus with our additional scholarly knowledge of mental health policy and service systems during data interpretation.

## Findings

Impressions of the most significant challenge in MC work varied across and within participant responses, with nearly half (45.6%) of participants identifying multiple challenges.

We identified five domains of challenge for MC workers. The five domains were (1) structural characteristics of MC work, (2) resources, (3) workplace and workforce, (4) provider mental health, and (5) behavioral health (BH) and criminal justice (CJ) systemic factors (See Table 2 for summary). Each direct quote utilized represents a distinct respondent.

**Table 2:** Clustered Summary Table of Challenges Faced by Mobile Crisis Workers

Challenge Domain (count)	Definition and Dimensions	Illustrative Quotes
*Structural Characteristics of Mobile Crisis Work (165)*	Challenges stemming from the temporal, spatial, and serious nature of mental health crises and MC intervention, like severity, urgency, variability, community location, and provider independence Dimensions: brief provision of intervention, unpredictability and uncertainty, decision-making, safety and violence, treatment refusal and avoidance, uncoordinated mental health services and related services, and difficult interactions with service user kin	“People are different – not everyone can be dealt with in the same fashion. You must think on your feet and be ready for anything.” “[The challenge is] the grey area when clients don’t meet criteria for an involuntary hold but are still unstable and at high risk”
*Resources (124)*	Challenges pertaining to resource availability and access, including material and service resources but excluding policies related to expenditures Dimensions: lack of service or resource, accessibility of service or resource, availability of service or resource	“Finding resources quickly for those who are in crisis situations” “Not enough facilities in the area at times that have beds available especially for children and people who have a co-occurring substance abuse disorder and need detox along with psychiatric stabilization”
*Workplace & Workforce (110)*	Challenges related to the workplace, agency where the crisis worker is employed, or the broader labor force, including administrative policies, procedures and employee dynamics Dimensions: shifts and hours, staffing and staff support, training, and leadership or management	“Inconsistency in how crisis workers approach a scenario due to a lack of training” “Low pay, and low staffing levels because of the low pay” “Long hours and little appreciation for the work we do”
*Provider Mental Health (91)*	Challenges regarding the relationship between provider mental wellbeing and mobile crisis work Dimensions: burnout and stress, vicarious trauma, doubt and guilt, and sleep disturbance	“The overall emotional toll of empathy for persons in crisis situations” “High stress from taking [on] the load of others to be able to provide appropriate care” “Worry that I will make the wrong decision, and someone will be harmed as a result”
*Behavioral Health (BH) and Criminal Justice (CJ) Systemic Factors (77)*	Challenges related to policies, procedures, system continuity in the broader political and historical context Dimensions: misalignment with collaborating entities, system discontinuity, federal and state laws, and funding structures	“The most challenging thing about being a mobile crisis worker is understanding the processes. Overcoming the barriers of the judicial system. Working with physicians in ED who aren’t familiar with mental health.” “We sometimes don’t understand why police do what they do, and they don’t understand our job and all the barriers that we have to jump over.” “The United States and individual states need to place more emphasis on funding mental healthcare and having a place for people to go when they are experiencing a crisis. Also making it so that the average person can AFFORD healthcare before they have a crisis”

Note. Each direct quote utilized represents a distinct respondent. “Count” is used to indicate the number of unique mentions that identified the associated theme

## Structural Characteristics of Mobile Crisis Work

The most common challenges participants identified were those related to structural characteristics of mobile crisis work (165 unique mentions). Specifically, these challenges related to the:

- Temporality of mobile crisis work, i.e., the urgent, fast-paced, and time-limited nature of MC crisis response.
- Spatiality of mobile crisis work, i.e., the home or community-based and broad geographic contexts of MC work.
- Seriousness of high-stakes situations prevalent in MC work, i.e., where service users are often experiencing severe distress and intervention decisions and outcomes can have life altering consequences.

We identified seven dimensions of structural characteristics that illustrate *how* the temporality, spatiality, and seriousness of MC work challenges service delivery. For example, participants spoke of challenges related to the brief provision of intervention, the urgency of the situation, and the need to make critical intervention decisions with often time-limited options. Crisis workers also described how the temporal, spatial, and serious nature of MC work combined to exacerbate each other and result in a sense of uncertainty, unpredictability, and idiosyncrasy that made crisis situations challenging to navigate. One participant explained that:

[I]n any given crisis you are not exactly sure what you will be walking into and if your team and/or the consumer will be in danger. No situation is black and white so it is hard to prepare yourself for what you might encounter because each case is so unique.

The seriousness of MC work referenced in the quote above also contributes to difficulty with decision-making under temporal constraints, as another participant explained, “it’s challenging knowing the exact tools, words to say, and decisions that will most effectively help a person in crisis.” The temporal, spatial, and serious nature of MC work means that team members often do not have pre-existing relationships or established trust with service users, resulting in reluctance to receive a higher level of care. One participant noted, it is challenging “when clients do not want a higher level of care and are very scared, but they are without a doubt unable to care for themselves safely.” Relatedly, time-limits, severity, and spatiality—where workers are quickly assessing circumstances and are often in someone’s home — put MC workers in situations where they had to manage family member expectations of mobile crisis workers and interventions available. To a lesser extent, MC workers described challenges related to safety, such as the unpredictable behavior of consumers.

## Resources

Crisis workers commonly described resource-related challenges (124 unique mentions), including resource scarcity, nonexistence, or inaccessibility. Participants highlighted that service users often need to be connected to other services or have co-occurring needs, but that it is impossible to make such connections or address these needs in the absence of a robust and coherent mental health service and support system. Specific resources that were lacking included those to meet the consumer’s basic needs (e.g., food, cash assistance), inpatient or community-based mental health services (e.g., crisis stabilization, detox/sobering facilities), housing, and non-law enforcement facilitated transportation. Relatedly, crisis workers identified challenges pertaining to extensive waitlists or limited hours of operation across these resource categories, while others were challenged by “not always having [other] resources for the person and their love[d] ones to access such as housing, food, [and] shelter.” Another participant explained, there are “not enough resources available…includ[ing] things to link clients to (outpatient therapy, psychiatry, housing supports, etc.) and also our own mobile response (the wait time is way too long).” Like the response above, participants commonly discussed limitations across multiple resources, which together hindered the provision of care.

## Workplace & Workforce

Participants described challenges that started within their agencies, the structure of their work, and in the terms of their employment (110 unique mentions). They expressed difficulty with the structure of shifts and hours, with one participant recounting:

I was on call Sunday night and was out until 1:30 AM. I had to write notes from the calls, so I wasn’t able to go to bed until 4:30 AM. I was up at 6:30 so I could be at work at 8:30 AM. This happens more often than not.

Participants also frequently described staffing problems related to the quantity and quality of workers, and lack of training, stating that there are “not…enough experienced and well-trained crisis workers on my team and in the community” and that it’s challenging “working with inexperienced crisis workers with zero background in [mental health], substance use, and the field in general.” Other less mentioned workplace and workforce challenges included lack of mental health supports for MC workers, inadequate leadership and management of the agency, insufficient wages, and logistical challenges specific to the agency or coverage area (e.g., the inconsistent volume of crisis calls, rural coverage area issues).

## Provider Mental Health

Crisis workers spoke of several ways in which their work negatively impacted their mental health (91 unique mentions). The two most common dimensions regarding provider mental health were burnout and stress or vicarious trauma. Participants referred to “crisis fatigue,” “empathy fatigue,” “desensitization to crisis,” “moral injury” and “high stress from taking the load off others.” Participants identified vicarious trauma and spoke to the general challenge of “hearing some of the heartbreaking stories and situations these individuals have experienced,” as well as mental health challenges related to witnessing disturbing incidents (e.g., child abuse) on the job. For some, the effect was described as feelings of hopelessness or sadness; for others, feelings of doubt, guilt and worry related to intervention decisions and outcomes, weighed on them. Participants explained the discretionary nature of the work was a challenge to their mental health because the stakes felt high, relaying they “worry [they] will make the wrong decision and that someone will be harmed as a result” and they struggle to handle “the pressure of making the wrong decision or making the right decision and someone…still end[ing] their life.”

### Behavioral Health (BH) and Criminal Justice (CJ) Systemic Factors

Crisis workers expressed that interaction and confrontation with inadequate or unsupportive systems, policies, and funding systems impeded MC work (77 unique mentions). Of these BH and CJ systemic factors, participants most frequently mentioned issues with law enforcement and the criminal legal system. For example, participants found it difficult to work with “police officers who aren’t trained properly or provide little support to crisis workers.” Other systemic challenge dimensions included misuse and misunderstanding of MC services, and difficulty ensuring adequate continuity of care between systems and services for service users. For example, a crisis worker noted:

[W]hen I do take individuals to the emergency room, I am not always sure that they are going to be admitted. I may have just brought a highly delusional, suicidal person, and we receive a call for service an hour later as they are out in the community again. I also do not have a homeless shelter or alternative housing, which means many of our [seriously mentally ill] are homeless and on the streets. Most of the calls for them are nuisance calls and not mental health related.

Thus, a lack of systemic continuity, access to alternative mental health services, and understanding of criteria for involuntary hospitalization interacted to impair service delivery.

### Additional Challenges of Mobile Crisis Work

Some participants identified challenges that fell outside of the five domains described above, two of which are worthy of note. First, a significant minority spoke to a general sense that the MC worker is unable to effectively help people; this challenge was noted to be distinct from responses that mentioned a specific impact of this perceived inability on their own mental health. Second, a small minority of participants identified a challenge related to the presentation of the service user. These challenges included active substance use, psychosis, or attitude towards crisis workers – i.e., “clients who are resistant to making changes” and trying to “help clients [who] struggle to help themselves”; “working with psychotic patients”; “dealing with substance users who don’t realize they are in crisis”; and, “when clients will not deescalate or separate from something they have that will hurt them.” MC workers’ minimal identification of service user presentation as challenging indicates that MC workers are less concerned directly with fear of the service user but rather a lack of adequate training and resources to best handle a variety of BHCs.

## Discussion

Recent policy trends, like the establishment of the 988 Suicide & Crisis Hotline, have demonstrated increased interest in improving crisis service infrastructure on a national level. Although MC teams and response services constitute a key component of comprehensive crisis systems, little research has identified challenges to the quality or sustainability of the MC workforce. This qualitative study revealed an array of challenges across five primary domains. Below, we discuss study limitations and summarize and contextualize key findings in prior research. We then translate the key findings into recommendations for improvements that can better sustain the MC workforce and therefore improve the capacity of MC services to aid those experiencing BHCs.

Our findings indicate that MC workers experience significant challenges to MC service delivery in the following domains: (1) structural characteristics of mobile crisis work, (2) resources, (3) workplace and workforce, (4) provider mental health, and (5) behavioral health (BH) and criminal justice (CJ) systems and structures. These findings are consistent with related literature on challenges experienced in the mental health workforce and other social service systems.

Within the broader mental health field, difficulties with provider decision-making around serious mental health interventions, particularly in the case of involuntary hospitalization, are well-documented from a variety of service settings around the world (Laureano et al., 2024). Similar to the experiences described by survey participants, these difficulties have been found to be multifaceted, involving ethical concerns, provider-family dynamics, and unclear intervention criteria (Laureano et al., 2024). Resource scarcity is also not unique to the mobile-crisis context as it is well-established that the United States’ mental healthcare system is severely underfunded with mental healthcare spending comprising only 5.6% of total US healthcare expenditures despite calls for parity between physical and mental healthcare (Hernandez & Uggen, 2012; Mahomed, 2020; Weil, 2015). Mental health resource constraints raise concerns about the sustainability of already overburdened agencies and workers as 988 hotline use continues to increase (Fix et al., 2023).

These concerns are not unique to MC response, as previous research has documented the relationship between workforce challenges and mental health among other types of first responders (Bolzon & Nalmasy, 2021). Mobile crisis worker descriptions of the significant impact of crisis work on their mental health are consistent with broader first responder and mental healthcare worker literature that cites experiences of burnout secondary to workforce and resource shortages, particularly in the wake of the COVID-19 pandemic (Bolzon & Nalmasy, 2021; Butryn et al., 2017). The perceived interaction between provider mental health and workforce or resource shortages is likely further compounded by the documented impact of structural inadequacies, such as poor funding, policy shortfalls, and disparate service accessibility (Ballout, 2025; Odes et al., 2024).

Though many of the challenges we identified align with challenges identified in prior research among adjacent service providers, we found important nuance in the way the temporality, spatiality, and seriousness of MC work overlapped and interacted with the other domains of challenge. Notably, we found that the latter four domains were most often described by participants in the context of resulting from or being related to the structural characteristics of mobile crisis work domain. For example:

- Burnout and stress experienced by the provider (provider mental health) often was reported to result from consistently seeing service users in a severe state of crisis (structural characteristics of MC work).
- Pressure to pursue involuntary hospitalization due to the temporal urgency and seriousness of the crisis (structural characteristics of MC work) was perceived to be exacerbated by scarcity of alternative community mental health resources (resources).
- The uncertain and unpredictable nature of MC work and high-stakes decision-making (structural characteristics of MC work) were reported to be made more challenging by lack of training and unclear agency policies (workplace and workforce).

The observation that challenges often are perceived to overlap and interact to worsen MC work conditions, as described above, is more than a conceptual abstraction; it suggests that effective MC team interventions and a sustainable workforce require innovations and reforms not only in MC interventions themselves, but also across service systems and policy arenas.

Although there is no easy solution nor silver bullet for these challenges, the first-hand perspectives of MC workers and existing literature suggest four priorities for advancing MC crisis teams, MC work, and building a sustainable workforce.

### Priority 1. Implement a Trauma-informed Mobile Crisis Work Environment

MC workers frequently described the fast-paced, severe, intense, and unpredictable nature of their jobs as detrimental to their mental health and ability to effectively provide care to service users. Proactive implementation of trauma-informed workplace settings and managerial supervision can more holistically support crisis workers by addressing MC worker mental health to mitigate the effects of vicarious trauma (Plouffe, 2015). More specifically, trauma-informed workplace settings can mitigate many of the challenges described by MC workers, such as feeling unsupported at work and struggling with compassion fatigue, by promptly responding to their needs early (Choitz & Wagner, 2021). As MC teams tend to incorporate significant supervision into their agency structure, Kim et al. (2021) recommend ensuring the commitment of leadership and supervisors to support top-down implementation of a trauma-informed workplace (2021). It is critical that trauma-responsive behaviors are integrated into the organization’s policies and procedures (see, e.g., SAMHSA, 2014, 2023), as well as modeled by those in leadership positions at the agency to showcase to staff and provide opportunities to practice the actionable impact of a trauma-informed workplace (Esaki, 2020). Supervisors should be trained to facilitate open dialogue among staff, conduct formal and informal debriefs following particularly challenging crisis calls, and foster an environment in which seeking therapeutic support as a crisis worker is openly supported (Kim et al., 2021). Additionally, agencies should continue to integrate mental health screenings with frontline workers, explore evidence-based trauma-informed trainings with all agency staff to prevent and quickly identify signs of burnout or early mental health concerns that may lead to employee attrition if left unaddressed (Bolzon & Nalmasy, 2021), and provide adequate resources such as sufficient paid time off for staff to be able to practice self-care (Sutton et al., 2022).

### Priority 2. Clarify Agency-level Policies, Procedures, and Standards

The unpredictability and uncertainty highlighted by MC workers was often found to correlate with mention of unclear policies, procedures, or standards for work at their specific agency. We recommend that service leaders in the field begin with the comprehensive steps outlined in “Roadmap to the Ideal Crisis System” published by the National Council for Mental Wellbeing and tailor the proposed action items to the constraints of each agency to formulate effective policies and procedures (2021). Engaging with existing staff, experts, and key stakeholders (e.g., service users and community leaders) is also recommended for best practice in developing protocols. Standardization and clarification of policies and procedures at agencies providing MC services could also improve the quality of measurable data and ease evaluation through reliable research to create a stronger feedback loop for the field of mental health crisis response (Muehsam, 2019; National Council for Mental Wellbeing, 2021). Additionally, as a compliment to this study, expanding research efforts to include MC worker perspectives on the rewards or benefits of MC work is encouraged to better understand successful service components and scalable opportunities at the agency-level. Consistent with Priority 1, this recommendation provides a ready-made opportunity to increase integration of trauma-informed care into agency policies.

### Priority 3. Develop Training Standards and Improve Mobile Crisis Worker Role Clarity

MC workers continue to identify the lack of standardized training as inhibitory to effective service provision. To be clear, we are not advocating for increased professionalization and inaccessibility of the field through required degree programs, but rather facilitating standardized practical application or certificate-awarded training for those working in MC. Trainings should be shaped by existing literature on crisis response and intervention (see, e.g., National Council for Mental Wellbeing, 2021), and focus on practical skills for assessment of circumstances, needs, and interventions as they pertain to service users across a variety of BHC. Comprehensive training also can improve role division and clarity between MC teams and collaborating agencies, such as law enforcement (Bailey et al., 2018). It is our recommendation that these training standards be established, and that agencies review and expand detailed role descriptions for MC workers to increase feelings of certainty and confidence regarding job responsibilities. Agencies should engage directly with local law enforcement and other collaborating agencies to facilitate mutually beneficial discussions regarding the roles of each staff member on the scene of a crisis, in relation to each other, and in the surrounding community.

Additionally, the continued growth of the peer support workforce as a subset of the mobile crisis workforce cannot be ignored - the significant group of survey participants (36.59%) who identified as peers is a testament to this. However, only 10.30% indicated their title to be MC peer advocate. This discrepancy reveals the need for further investigation into the experience of MC workers who are navigating their peer identity both as an official and unofficial part of the job. Furthermore, improved role clarity is critical to ensuring providers across the continuum understand the unique perspectives, skills, and capabilities of peers (Tham et al., 2025). To support this effort, further research is needed to better understand the first-hand experiences of peers working in mobile crisis, including how the challenges of mobile crisis work may be distinctly defined or experienced by this population of workers.

### Priority 4. Allocate Adequate Funding Towards Community Mental Health and Broader Social Welfare Systems

The difficulty expressed by MC workers with attempting to support service users while navigating a lack of community mental health resources is a testament to the need for stable funding allocation towards bolstering mental health service systems (Odes et al., 2024). While implementing 988 was a crucial step forward, expansion of federal grant programs specifically targeted towards uplifting crisis response services would demonstrate a stronger commitment to improving nationwide mental health support (Hogan & Goldman, 2021). Increased funding through grant opportunities and revised reimbursement codes could combat the existing reliance on involuntary hospitalization described by crisis workers through allowing development of alternatives, such as outpatient services, community crisis stabilization beds, and peer support (Marcus & Stergiopoulos, 2022; National Council for Mental Wellbeing, 2021; Pietras & Wishon, 2021). Furthermore, MC workers drew attention to the fact that many MC service users were simultaneously experiencing housing instability or economic hardship; thus, continuing to bolster social welfare systems through funding of options such as affordable housing and cash and basic needs assistance programs is integral to well-rounded support in addition to MC services.

### Limitations

Though this study provides important insights on MC worker perspectives and challenges in mobile crisis work, several limitations are worthy of note. Because a uniform organization or list of agencies currently providing MC services to the community on a national scale does not exist, we could not use probability sampling and instead relied on an availability sample of MC workers. We attempted to maximize sample diversity by utilizing varied recruitment methods and targeting agencies in all 50 states. Ultimately, however, we received responses from only 24 states and cannot rule out the possibility that certain subsets of MC workers (e.g., CIT workers and MC supervisors) are overrepresented because they were more available or amenable to participation in the study and because no national data exists on this workforce for comparison. Lastly, since this study relied on analysis of narrative text-form responses provided to open-ended questions via an anonymous, confidential online survey, findings were authenticated through researcher triangulation and thorough refinement of trends into themes. Clarifying follow-up information that might have been gained in an interview or focus group format was unavailable to the research team. The method we utilized, however, does permit crucial understanding and description of complex phenomena that can then be considered, alongside existing literature, when applying knowledge gained from the current study to other related situations (Polit & Beck, 2010); thus, this study was able to provide concrete recommendations drawn from MC workers and supervisors to improve MC service provision.

## Conclusion

According to a national sample of MC workers, challenges to MC service delivery are numerous and complex, leaving many MC workers feeling as though they are “acting as the face of a broken system.” We found that these challenges reportedly stemmed from the structural characteristics of MC work: temporality, spatiality, and seriousness. However, we determined these challenges were often perceived to be exacerbated, effected, or compounded by issues pertaining to resources, the workplace and workforce, provider mental health, and BH and CJ systemic factors. Because of this pattern, we suggest going beyond the search for one single solution and recommend policymakers and administrators seek to enact change across levels, from individual agencies to social and mental health services and policies. To improve the capacity of MC teams and better support crisis response systems, comprehensive changes to workplace environments, administrative policies, training standards, interagency and interprofessional collaboration, and social welfare and mental health service systems must be prioritized. As policymakers and community mental health providers attempt to fulfill the promises of prior legislation and meet the growing needs of communities, we hope they will act on these recommendations to build a well-functioning mental health service system that MC workers are proud to represent.

## Data Availability

No datasets were generated or analysed during the current study.

## Footnotes

1 The list of mobile crisis organizations by state compiled in 2020 by Keyne Law, PhD at Seattle Pacific University has not been previously published and can currently be found on X (Twitter): Law, K. [@keyneclaw]. (2020, October 19). *US mental health providers and researchers*,* if you or your clients are uncomfortable calling 9-1-1 and/or involving law enforcement during* [Post]. X. https://x.com/keyneclaw/status/1318307561251106816?s=46.
